# Modified Peptide YY Molecule Attenuates the Activity of NPY/AgRP Neurons and Reduces Food Intake in Male Mice

**DOI:** 10.1210/en.2019-00100

**Published:** 2019-05-10

**Authors:** Edward S Jones, Nicolas Nunn, Adam P Chambers, Søren Østergaard, Birgitte S Wulff, Simon M Luckman

**Affiliations:** 1 Faculty of Biology, Medicine and Health, University of Manchester, Manchester, United Kingdom; 2 GLP-1 & T2D Pharmacology, Novo Nordisk A/S, Novo Nordisk Park, Måløv, Denmark; 3 Research Chemistry 2, Novo Nordisk A/S, Novo Nordisk Park, Måløv, Denmark; 4 Obesity Research, Novo Nordisk A/S, Novo Nordisk Park, Måløv, Denmark

## Abstract

To study the effects of an analog of the gut-produced hormone peptide YY (PYY_3-36_), which has increased selectivity for the Y2 receptor; specifically, to record its effects on food intake and on hypothalamic neuropeptide Y/agouti-related peptide (NPY/AgRP) neuron activity. NNC0165-1273, a modified form of the peptide hormone PYY_3-36_ with potent selectivity at Y2 receptor (>5000-fold over Y1, 1250-fold over Y4, and 650-fold over Y5 receptor), was tested *in vivo* and *in vitro* in mouse models. NNC0165-1273 has fivefold lower relative affinity for Y2 compared with PYY_3-36_, but >250-, 192-, and 400-fold higher selectivity, respectively, for the Y1, Y4, and Y5 receptors. NNC0165-1273 produced a reduction in nighttime feeding at a dose at which PYY_3-36_ loses efficacy. The normal behavioral satiety sequence observed suggests that NNC0165-1273 is not nauseating and, instead, reduces food intake by producing early satiety. Additionally, NNC0165-1273 blocked ghrelin-induced cFos expression in NPY/AgRP neurons. *In vitro* electrophysiological recordings showed that, opposite to ghrelin, NNC0165-1273 hyperpolarized NPY/AgRP neurons and reduced action potential frequency. Administration of NNC0165-1273 via subcutaneous osmotic minipump caused a dose-dependent decrease in body weight and fat mass in an obese mouse model. Finally, NNC0165-1273 attenuated the feeding response when NPY/AgRP neurons were activated using ghrelin or more selectively with designer receptors. NNC0165-1273 is nonnauseating and stimulates a satiety response through, at least in part, a direct action on hypothalamic NPY/AgRP neurons. Modification of PYY_3-36_ to produce compounds with increased affinity to Y2 receptors may be useful as antiobesity therapies in humans.

Obesity is a major public concern, and common associated comorbidities such as type 2 diabetes mellitus and heart disease place enormous health, productivity, and financial burdens on society (1). To date, there has been a lack of clinical-grade pharmaceutical interventions to target the growing obesity crisis, with only a select few medications currently available, such as lorcaserin and liraglutide, that produce modest body weight–lowering effects (2). One hope is to adapt naturally occurring gut-produced hormones that may act as satiety factors, such as shown for GLP-1 analogs such as liraglutide (3).

Peptide YY (PYY) is a 36-amino acid protein that is coproduced with other gut hormones throughout the length of the intestine, although levels are higher in more distal regions (4). It is secreted postprandially in response to luminal nutrients and acts locally within the gut to regulate pancreatic and gastric secretion (5) and gastrointestinal motility (6). PYY is a member of the neuropeptide Y/pancreatic polypeptide family and has high affinity for shared receptors (Y2 > Y1 > Y5 > Y4 receptors). However, secreted PYY is rapidly converted by dipeptidyl peptidase-4, so that the major circulating hormone is the truncated form, PYY_3-36_ (7). This peptide retains its high affinity for Y2, but has reduced affinity for both Y1 and Y4, and around a 10-fold reduced selectivity for Y5 (8, 9), and has multiple effects on energy balance including control of satiety and gut motility (10).

The first indication that PYY_3-36_ can act as a circulating factor to reduce food intake and body weight came from Batterham *et al.* (11), and this has since been corroborated by other studies in rodents (12–17) and nonhuman primates (18, 19). Administration of exogenous PYY_3-36_, to produce physiological postprandial concentration in the blood, reduces food intake and increases energy expenditure in both lean and obese humans (20, 21). These effects of PYY_3-36_ are believed to be mediated primarily by binding to Y2 receptors on neurons of the mediobasal hypothalamus, which are open to circulating hormones. Indeed, direct injection of PYY_3-36_ into the rodent hypothalamic arcuate nucleus results in a reduction in feeding behavior (22), compared with injection into the brain ventricular system, which causes the opposite effect (23), the latter probably resulting from local activation of the Y5 receptor.

Inhibitory Y2 autoreceptors are located on arcuate neuropeptide Y/agouti-related peptide (NPY/AgRP) neurons as well as other neurons in the mediobasal hypothalamus; if activated, these will have anabolic effects (increased energy intake and decreased energy expenditure). Thus, neuronal inhibition via Y2 receptors in the mediobasal hypothalamus is an obvious, though still unproven, target for postprandial, blood-borne PYY_3-36_. On this note, other studies have suggested that the anorectic effects of PYY_3-36_ are vagally mediated (24) or, instead, that it induces nausea, conditioned-taste aversion, and/or altered taste perception by acting through the brainstem (25–27).

Whatever its mechanism of action, it is sufficiently different to that of other gut hormones that the most promising treatments may be cotherapies capable of producing synergism. Thus, administration of PYY_3-36_ plus either glucagon-like peptide 1 (28), oxyntomodulin (29), or pancreatic polypeptide (29) is more effective at producing weight loss than the individual monotherapies. However, an issue remains, because PYY_3-36_ still has important affinity for Y1 and Y5 receptors, which have confounding actions to increase food intake centrally (30) and to cause fat accretion peripherally (31, 32). Taken together, a treatment that biases agonism at hypothalamic Y2 receptors over Y1 and Y5 receptors could be more effective at reducing food intake without increasing adiposity.

Here we describe a modified PYY_3-36_ molecule, NNC0165-1273, with at least 650-fold greater affinity for Y2 over any other receptor. Using this compound, we demonstrate that acute administration to mice reduces both normal nighttime feeding, and ghrelin-induced feeding, concomitant with shortening the behavioral satiety sequence. The peptide is more efficacious than PYY_3-36_ in these scenarios. Longer term treatment of diet-induced obese (DIO) mice, for 2 weeks, caused a sustained reduction in food intake and body weight. Electrophysiological recordings *in vitro* demonstrated a direct inhibitory effect on NPY/AgRP neurons in the arcuate nucleus. Finally, using ghrelin or chemogenetic stimulation, we demonstrate that NNC0165-1273 can attenuate the direct activation of NPY/AgRP neurons in the mediobasal hypothalamus, providing further evidence for this neuron being a major Y2 target.

## Materials and Methods

### Peptides

PYY_3-36_ and ghrelin were purchased from Bachem (Bubendorf, Switzerland). NNC0165-1273 was identified from a series of modified PYY peptides and synthesized with the aim of increased selectivity for Y2 receptors vs the other Y receptors [peptide no. 29 in Østergaard *et al.* (9)]. NNC0165-1273 showed a binding inhibitory constant (K_i_) on the human Y_2_ receptor of 2 nM and >10,000, 2500, and 1300 nM K_i_ values on the human Y1, Y4, and Y5 receptors, respectively. This compared with a K_i_ of PYY_3-36_ of 0.40 nM on the Y2 receptor and 40, 13, and 3.2 nM on the Y1, Y4, and Y5 receptors, respectively, when tested in the same binding assays. This indicates a fivefold lower relative affinity of NNC0165-1273 for the Y2, but >250, 192-, and 400-fold selectivity, respectively, for the Y1, Y4, and Y5 receptors. Additionally, in *in vitro* metabolism studies, the peptide has as a half-life of 570 minutes vs 200 minutes for PYY _3-36_ as measured in pig plasma. [For further information, description of the peptide and methods, see Østergaard *et al.* (9).]

### Mice and surgery

All experiments were performed in accordance with Home Office animal use regulations, the EU Directive 2010/63/EU for animal experiments, and local ethical review. Adult male mice were maintained on a 12 hour/12 hour light/dark cycle, with unlimited access to standard chow (Special Diet Services, Essex, UK) and water. For DIO, C57BL/6 mice (Charles River, Wilmington, MA; starting at 4 to 6 weeks of age) were placed on *ad libitum* 60% high-fat diet (D12492; Research Diets, New Brunswick, NJ) for 4 months before arriving at the test facility, at which point mice were switched to a 45% high-fat diet (HFD D12451; Research Diets) to prevent crumbling and spillage and more accurately measure food intake. *AgRP-*cre (no. 012899) (33) and *Npy-*hr green fluorescent protein (GFP) (no. 006417) (34) mice were obtained from Jackson Laboratories (Bar Harbor, ME).

For surgery, mice (8 to 12 weeks) were anesthetized with isoflurane (1 L/min O_2_ with 2% isoflurane). Following surgery, all mice received 0.03 mg/kg buprenorphine (Buprenex, Reckitt Benckiser, Slough, UK) and were allowed a minimum of 1 week to recover before treatment started. DIO mice received subcutaneous implants of an Alzet 2002 osmotic minipump (Durect, Cupertino, CA). All of the DIO mice experienced a small reduction in body weight following implantation of the minipump. For experiments using designer receptors exclusively activated by designer drugs (DREADDs), *AgRP*-cre mice received bilateral injections of AAV2-hSyn-DIO-hM3Dq-mCherry (UNC Vector Core, Chapel Hill, NC) (35) into the arcuate nucleus (3 × 23 nL per side), using the following coordinates relative to Bregma, according to Paxinos and Franklin (36): anterior, −1.5 mm; lateral, ±0.30 mm; and ventral, −6.2 mm. Subsequently, the DREADDs were activated by IP injection of the designer drug, clozapine *N*-oxide (CNO; Sigma-Aldrich, St. Louis, MO) at 1 mg/kg.

### Feeding and behavior studies

For acute experiments, mice were acclimated to handling for 2 weeks and housed singly with free access to food at least 4 days before experiments. Nighttime injections of NNC0165-1273 were given subcutaneously (SC) 30 minutes before start of “dark phase,” and food intake was measured 1, 2, 4, and 14 hours later. Daytime feeding experiments commenced 4 hours into the “light phase” [Zeitgeber time (ZT) 4]; injections of ghrelin (2 mg/kg, IP) and NNC0165-1273/PYY_3-36_ (various doses, SC) were given concomitantly, and food intake measured at 1, 2, 4, and 24 hours later.

For long-term, mini pump experiments, DIO mice were scanned using a mouse-specific MRI machine (EchoMRI™-130, Zinsser Analytic GmbH, Eschborn, Germany) at the beginning and end of the experiment. Body weight and food intake were measured daily at 4 hours into the light phase (ZT 4) for 2 weeks during treatment. For DIO mice in the TSE system (TSE Systems, Bad Homburg, Germany), food and water intake, VCO_2_ and O_2_ were measured automatically every 9 minutes.

To monitor the behavioral satiety sequence (BSS), C57BL/6 mice were singly housed for at least 4 days, before being fasted for 16 hours overnight. Animals received SC injections at 3 hours into the light phase (ZT 3) and standard chow was returned to each cage 20 minutes later. The behavior of each animal was assessed every 90 seconds for the following 90 minutes. Behaviors were classified as feeding, drinking, active (clear movement), grooming, inactive (huddled, sprawled), and resting.

### Immunohistochemistry

Mice were transcardially perfused with chilled 0.9% NaCl followed by 4% paraformaldehyde in 0.1M phosphate buffer (Sigma-Aldrich). Whole brains were postfixed overnight and then dehydrated in 30% sucrose solution. Brains were flash frozen on dry ice, and then 30 µm sections were collected using a freezing sledge microtome and transferred to 0.1M phosphate buffer.

For chromogenic visualization of cFos, free-floating sections were incubated sequentially in rabbit anit-cFos primary [1:1000; SC-52; Santa Cruz, Dallas, TX (37)], biotinylated goat anit-rabbit secondary [1:500; BA-1000; Vector Laboratories, Cambridge, UK (38)], and streptavidin-biotin-peroxidase complex (1:500; RPN1051; GE Healthcare, Chicago, IL). This was combined with fluorescence staining for GFP or mCherry reporters; this required coincubation of sections with chicken anit-GFP [1:1000; 13970; Abcam, Cambridge, UK (39)] followed by donkey anit-chicken^ALEXA 488^ [1:1000; 703-545-155, Jackson ImmunoResearch, West Grove, PA (40)], or with rabbit anti-DsRed [1:1000, 632396, Clontech, CA (41)] followed by donkey anti-goat^ALEXA 594^ [1:1000; 705-585-147; Jackson ImmunoResearch, (42)]. Sections were mounted on slides before imaging on an Olympus BX51 upright microscope. Images were processed and analyzed using open-source FIJI software.

### Electrophysiology

*Npy*-hrGFP mice were euthanized by decapitation, and the brains rapidly removed and sliced on a vibratome (Campden Instruments, Loughborough, UK) in ice-cold oxygenated incubation artificial cerebrospinal fluid (aCSF) containing (mM): 95 NaCl, 1.8 KCl, 1.2 KH_2_PO_4_, 7 MgSO_4_, 26 NaHCO_3_, 0.5 CaCl_2_, 15 glucose, and 50 sucrose. Slices were perfused with room temperature recording aCSF containing (mM): 127 NaCl, 1.8 KCl, 1.2 KH_2_PO_4_, 1.3 MgSO_4_, 26 NaHCO_3_, 2.4 CaCl_2_, and 5 glucose. *Npy*-hrGFP neurons were visualized in 250-µm slices using an Olympus BX51 with inbuilt infrared video-enhanced differential interference contrast optics and GFP fluorescence optics. Neurons were patched using 7 to 10 MΩ pipettes containing (mM): 130 K-gluconate, 10 KCl, 2 MgCl_2,_ 10 HEPES, 0.5 EGTA, 2 K_2_ATP, and 0.5 NaGTP. Data were recorded on an Axoclamp 2A amplifier in bridge mode (Molecular Devices, San Jose, CA) and a CED 1401 A/D data acquisition interface (CED, Cambridge, UK) to give current clamp data at 30 kHz sampling frequency. NNC0165-1273 (Novo Nordisk, Maaloev, Denmark) was diluted in aCSF to a concentration of 50 nM and applied to perfusion chamber through a gravity-driven system. Effects were sampled with no current manipulations. All applications were timed for 3 to 5 minutes and perfusion flow was maintained at standard 1 to 2 mL/min^−1^.

### Statistical analyses

All analyses used parametric statistics and tests were performed using GraphPad Prism 7 (GraphPad Software, San Diego, CA). Current clamp data were acquired with Spike2 version 7 (CED, Cambridge, UK).

## Results

### NNC0165-1273 reduces food intake and body weight, and does not adversely affect the BSS

**Figure 1.**
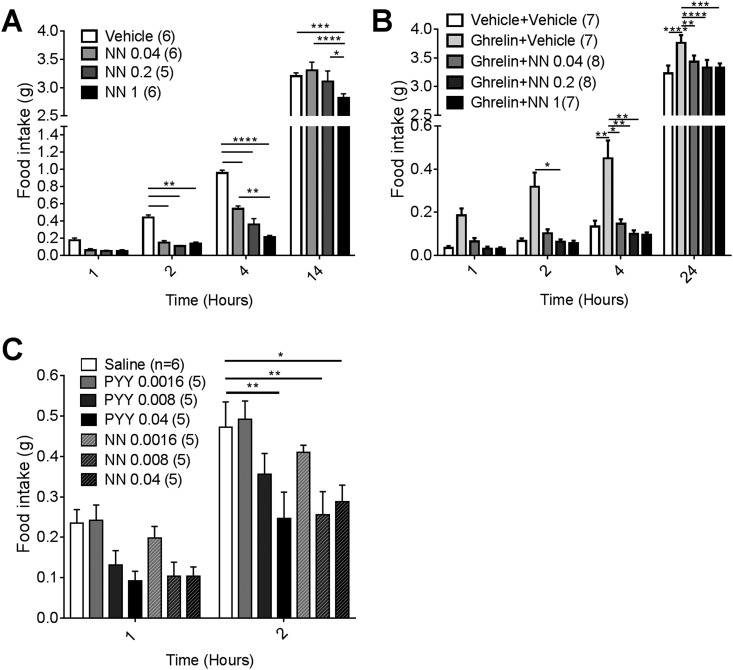
NNC0165-1273 reduces nighttime and ghrelin-induced feeding. The effect of 0, 0.04, 0.2 and 1 µmol/kg NN on cumulative (A) normal, nighttime feeding and (B) ghrelin-induced feeding during the light phase. NN was injected 30 minutes before lights out or concomitantly with ghrelin at 2 hours after lights on. (C) To compare the relative potency of NN and PYY_3-36_, lower doses of the peptides were injected SC and nighttime food intake measured. Data presented as mean ± SEM. Two-way ANOVA with Tukey *post hoc* tests: *P < 0.05; **P < 0.01; ***P < 0.001; ****P < 0.0001. NN, NNC0165-1273.

Mice received SC injections of either saline or one of three doses of NNC0165-1273 (0.04, 0.2, or 1 μmol/kg) 30 minutes before the start of the dark phase. All doses of NNC0165-1273 were effective, producing substantial reductions in food intake after 2 and 4 hours (Fig. 1A). To test the efficacy of NNC0165-1273 in suppressing ghrelin-induced feeding, mice received an initial IP. injection of either saline or ghrelin (2 mg/kg) followed by an additional SC injection of 0, 0.04, 0.2, and 1 µmol/kg NNC0165-1273. Ghrelin alone caused a substantial increase in food intake, and this was significantly attenuated by each dose of coadministered NNC0165-1273 (Fig. 1B). Because mice receiving 0.04 µmol/kg of NNC0165-1273 showed a marked reduction of food intake in both experiments, we used a serial dilution from that dose to find the threshold dose needed to inhibit nighttime feeding; this was performed in parallel with PYY_3-36_ at the same doses (Figure 1C). NNC0165-1273 significantly reduced food intake after 2 hours at 0.008 μmol/kg; by comparison 0.04 μmol/kg PYY_3-36_ was required to induce a substantial reduction in food intake (Fig. 1C).

**Figure 2.**
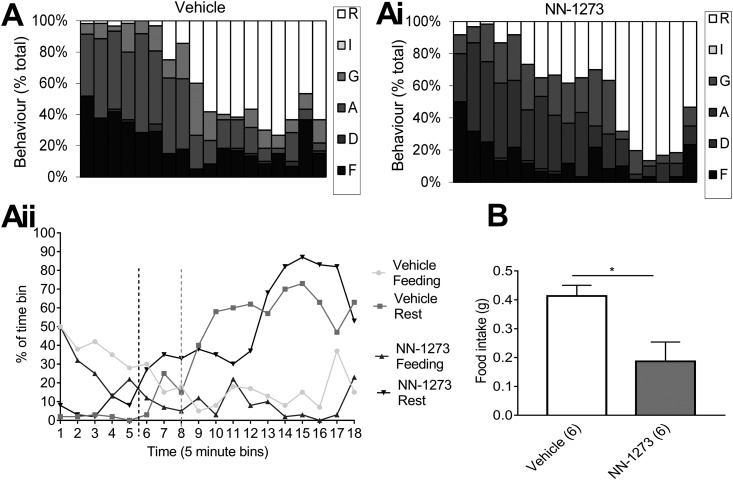
NNC0165-1273 advances satiety in the behavioral satiety sequence. Behavior was monitored in mice given food after injection of (A) vehicle or (Ai) 0.04 µmol/kg NNC0165-1273. Behaviors were classified as resting (R), inactive (I), grooming (G), active (A), drinking (D), and feeding (F). (Aii) To demonstrate the earlier satiety in NN-1273–treated mice, feeding and resting behaviors are plotted against each other. (B) At the end of the 90-minute observation period, food intake was measured. Data presented as mean ± SEM. Unpaired Student *t* test; *P < 0.01.

To test if NNC0165-1273 has any overt aversive effects on behavior, we plotted the BSS following a single injection of 0.04 µmol/kg of the peptide, compared with saline controls. Mice receiving the control injection progressed through the normal sequence of behaviors following the return of food to their cages: feeding, drinking, exploratory activity, grooming, and sleep (Fig. 2A). The mice receiving NNC0165-1273 showed exactly the same sequence (Fig. 2Ai), although shifted slightly over the 90-minute period, so the percentage of time spent feeding was reduced (22% vs 14%), and the percentage of time grooming and sleeping increased (12% vs 18% and 36% vs 40%, respectively). None of the mice presented with immobility or any other unusual behaviors. To visualize the progression of the BSS, we plotted the intersection of percentage times spent either feeding or sleeping in 5-minute time bins. For the control group, the transition between feeding and sleeping occurred in bin 8, and for the NNC0165-1273 group in bin 5, approximately 15 minutes earlier (Fig. 2Aii). At the end of the 90-minute period, the NNC0165-1273–treated mice had eaten significantly less food in comparison with controls (*P < 0.01) (Fig. 2B).

**Figure 3.**
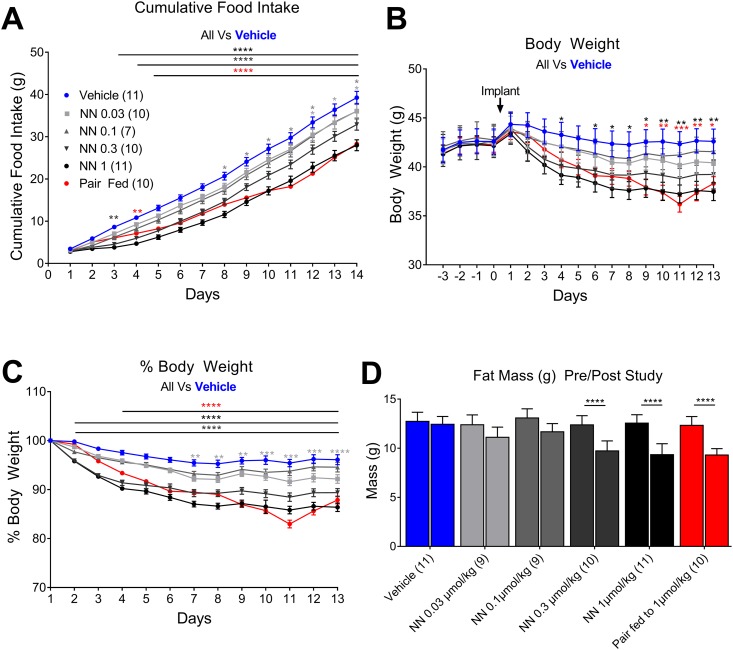
Infusion of NNC0165-1273 reduces both food intake and body weight in obese mice. There was a dose-dependent decrease in (A) cumulative food intake and (B) body weight over the 2-week period upon subcutaneous infusion with 0, 0.03, 0.1, 0.3, and 1 µmol/kg/d NN. (C) This was clearly apparent when the percentage body weight change was plotted against time. An additional control group, pair-fed to the 1 µmol/kg/d NN group, lost the same amount of weight. (D) Magnetic resonance scanning demonstrated that NN-1273 treatment caused a substantial decrease in fat mass. Data presented as mean ± SEM. Two-way ANOVA with (A–C) Dunnet and (D) Sidak *post hoc* tests: *P < 0.05; **P < 0.01; ***P < 0.001; ****P < 0.0001.

To determine the effects of long-term exposure of NNC0165-1273 on food intake and body weight, a dose-response experiment using administration via osmotic minipumps was performed. DIO mice received an infusion of NNC0165-1273 over a 2-week period, of 0, 0.03, 0.1, 0.3, or 1 µmol/kg/d. There was a dose-dependent decrease in cumulative food intake, body weight, and fat mass over the 2-week period (Fig. 3). An additional control group, pair-fed to the 1 µmol/kg NNC0165-1273 group, lost the same amount of weight/fat mass. Energy expenditure was also measured, with no important differences seen in VO_2_ or VCO_2_, although there was a substantial decrease in RER, suggesting NNC0165-1273 might be shifting the mice toward using fat as an energy source (43).

### NNC0165-1273 attenuates ghrelin-induced or chemogenetic-stimulated feeding via NPY/AgRP neurons

**Figure 4.**
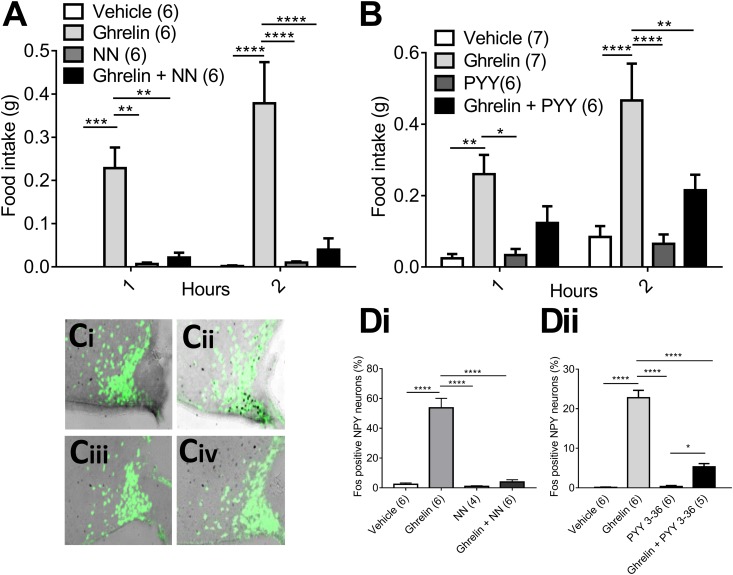
NNC0165-1273 blocks the feeding and cFos responses to ghrelin to a similar extent as PYY_3-36_. Preadministration of (A) NN (0.04 µmol/kg) or (B) PYY_3-36_ (0.012 μmol/kg) abrogated the feeding induced by a daytime injection of ghrelin (2 mg/kg), as well as the induction of cFos in NPY/AgRP neurons of the hypothalamic arcuate nucleus. Representative photomicrographs of fluorescent *Npy*-hrGFP neurons following injection of (Ci) vehicle, (Cii) ghrelin, (Ciii) NN, or (Civ) NN + ghrelin. (D) The percentage of NPY/Agrp neurons containing cFos following the same injections as in (A) and (B). Data presented as mean ± SEM. Two-way ANOVA with (A, B) Sidak *post hoc* tests: *P < 0.05, **P < 0.01, ***P < 0.001, ****P < 0.0001; one-way ANOVA with (D) Sidak *post hoc* test: *P < 0.05, ****P < 0.0001.

Injection of ghrelin (2 mg/kg) caused a substantial increase in food intake when injected during the light phase into fed mice. NNC0165-1273 (0.04 µmol/kg) had no important effect by itself, but was able to significantly attenuate food intake at 1 and 2 hours, when injected 1 minute before ghrelin (Fig. 4A). A similar result was obtained when using native PYY_3-36_ [50 µg/kg (*i.e.,* 0.012 μmol/kg)] to attenuate ghrelin-induced feeding (Fig. 4B). Mice were transcardially perfused with fixative 90 minutes after injection of ghrelin (2 mg/kg) to allow immunohistochemistry for cFos protein as a marker for neuronal activity. As predicted, ghrelin caused a substantial increase in the expression of cFos in the arcuate nucleus (44), notably in NPY-hrGFP neurons (Fig. 4C–4D). The activation of NPY/Agrp neurons was blocked by pretreatment with either NNC0165-1273 or PYY_3-36_ (Fig. 4C–4D).

**Figure 5.**
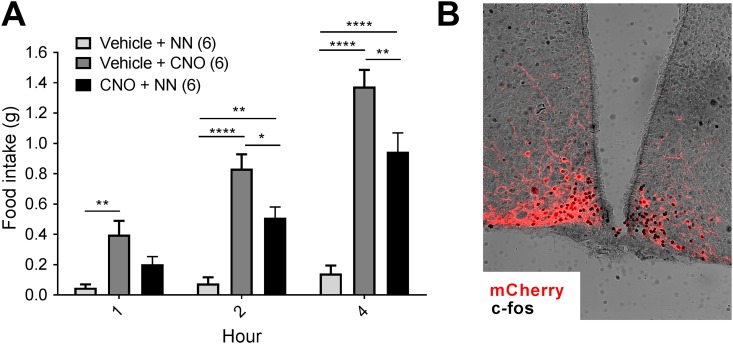
NNC0165-1273 partially blocked the stimulation of feeding by DREADD activation of NPY/AgRP neurones. NPY/AgRP neurons were targeted by bilateral injection of AAV-hM3Dq-mCherry into the arcuate nucleus of *Agrp*-cre mice. (A) Subsequently, direct activation of NPY/AgRP neurons by injection of the designer drug CNO (1 mg/kg) caused a substantial increase in daytime food intake, which was blocked by preadministration of NN (0.04 µmol/kg). (B) Representative example showing cFos in AgRP-hM3Dq neurons following activation with CNO. Data presented as mean ± SEM. Two-way ANOVA with Tukey *post hoc* tests: *P < 0.05; **P < 0.01; ****P < 0.001.

Because ghrelin can potentially induce feeding through a variety of pathways, we next looked at the action of NNC0165-1273 on NPY/AgRP neurons that had been activated directly through cell-specific designer receptors. *Agrp*-cre mice were successfully transfected in the arcuate nucleus with an AAV containing cre-dependent hM3Dq-mCherry. A single IP injection of CNO to *Agrp*-cre::hM3Dq-mCherry mice caused a substantial increase in food intake compared with saline controls (Fig. 5A), as observed previously (35). Because of some suggestions that CNO could, by itself, cause nonspecific pharmacological effects (45), we confirmed that wild-type mice do not respond to CNO with any change in food intake (results not shown). Preadministration of NNC0165-1273 significantly attenuated the feeding response to CNO in *Agrp*-cre::hM3Dq-mCherry mice (Fig. 5A). One week following this experiment, all the mice received an injection of CNO and were transcardially perfused with fixative. This was used to assess transduction of NPY/AgRP neurons with hM3Dq-mCherry and to show that they are activated by CNO using cFos immunohistochemistry (Fig. 5B).

### NNC0165-1273 acts directly on NPY/AgRP neurons in the arcuate nucleus

**Figure 6.**
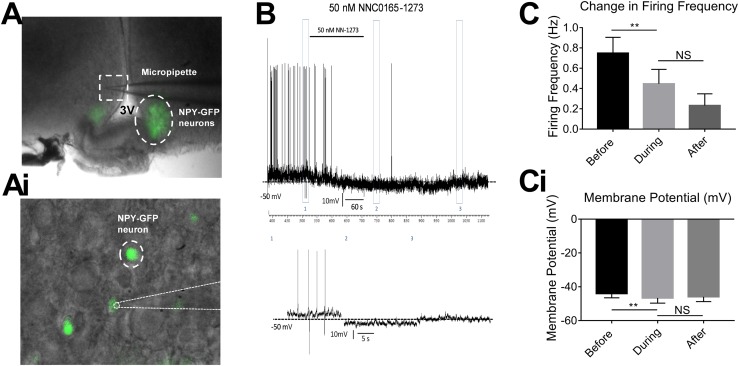
NNC0165-1273 reduced the firing frequency and membrane potential of NPY/AgRP neurons during current clamp patching of brain slices. (A) *Npy*-hrGFP expression is detectable in the arcuate nucleus. (Ai) Pipette attached to individual *Npy*-hrGFP neuron. (B) Example recording following 50 nM NNC0165-1273 application. (C) Changes in firing frequency and (Ci) in membrane potential (mV) before, during, and after 50 nM NNC0165-1273 application (n = 8). Data presented as mean ± SEM and analyzed using paired *t* tests. NS, not significant.

To demonstrate Y2 receptor agonism directly on NPY/AgRP neurons, we used patch-clamp electrophysiology in slices from *Npy*-hrGFP mice. Neuron activity was measured using a current clamp configuration and, as expected, bath application of 50 nM ghrelin was able to cause an increase in the resting membrane potential of NPY/AgRP neurons in the arcuate nucleus, sensitizing them to depolarizing stimuli (results not shown). NNC0165-1273 (50 nM) application significantly attenuated the firing frequency of spontaneously active arcuate NPY/AgRP neurons (Fig. 6C). NNC0165-1273 application also significantly reduced the membrane potential of arcuate NPY/AgRP neurons (Fig. 6Ci). Only in a minority of cases was action potential firing reinstated during the immediate washout period.

## Discussion

Here, we have shown that NNC0165-1273, a PYY_3-36_ analog with increased specificity to Y2 receptors, had potent effects on food intake and fat accumulation, with actions at the mediobasal hypothalamus. Our primary aim was to investigate the potential therapeutic value of NNC0165-1273 in the treatment of obesity. PYY_3-36_ is a potent anorectic peptide hormone released from the l cells in response to feeding (46) and acts at a number of the Y receptors, particularly Y1, Y2, and Y5 (47). Paradoxically, Y2 activity decreases food intake, whereas Y1 and Y5 activity increase food intake (11, 48). This, along with its short half-life, makes native PYY_3-36_ unappealing as a therapeutic. Therefore, a PYY_3-36_ analog, NNC0165-1273, which has at least 650-fold affinity for Y2 over the other Y receptors, as well as having a slightly increased *in vitro* half-life in pig plasma was developed (9). This should increase its potential as an obesity therapeutic by targeting the anorectic pathways with greater precision.

NNC0165-1273 had a potent anorectic effect in mice during normal nighttime feeding, as well as attenuating the stimulation of feeding by ghrelin, which recapitulates previous results shown for PYY_3-36_ (49). We further demonstrated that NNC0165-1273 required a lower threshold dose to produce a substantial reduction in feeding when compared with PYY_3-36_. This could reflect that, unlike NNC0165-1273, PYY_3-36_ has considerable activity via the orexigenic Y1 and Y5 receptors. Importantly, using a BSS, we showed that the anorectic effects of NNC0165-1273 were due to satiety rather than nausea, which is essential for therapeutic tolerance. This is notable because PYY_3-36_ induces nausea in humans in clinical trials and aversion in rodent studies (25, 50). In fact, this induction of nausea is a major limitation of PYY_3-36_ in a therapeutic setting. Understanding the receptors involved in this nausea response would be valuable when engineering antiobesity compounds as multiple anorectic peptides induce nausea at higher doses, including CCK, PYY_3-36_, and GLP-1. Whether a common circuit or system mediates this or if the nausea response can be induced by a number of separate peptide-neuron interactions remains to be seen.

Although central Y2 agonism is associated with a reduction in feeding and adiposity, peripheral Y2 agonism can produce opposing effects to accentuate adiposity via increased adipose tissue accretion (51). Simplistically, whereas Y2 receptors found in white adipose tissue are obesogenic, hypothalamic Y2 receptors are potently anorectic. Because PYY_3-36_ and NNC0165-1273 treatment reduce food intake over both the short- and longer terms, it appears as that the anorectic Y2 tone supersedes the obesogenic tone from white adipose tissue Y2, so the overall effect is weight loss primarily via a reduction of feeding.

Because Y2 is an inhibitory receptor, we would expect that a reduction in sympathetic tone would lead to increased adiposity. However, depending on the site of sympathetic innervation, level of tone, as well as the possible involvement and effects of *para*-sympathetic innervation, we may see a number of other important physiological changes. As such, it is difficult to predict the effects of peripheral Y2 agonism which is further confounded as these predictions may vary between animal models.

A common problem seen with candidate obesity therapeutic agents is attenuation of the response, resulting in partial or full reversal of weight loss during the therapy (2, 52). We therefore investigated the longer term effects of NNC0165-1273 by infusing it into obese mice over the course of 2 weeks and found highly substantial reductions in food intake, body weight, and fat mass in those animals, and no change in locomotor activity (53). There was no sign of any reversal of the weight loss during this time, which is encouraging for future translational studies. Pair-fed animals had the same body weight and fat mass changes as the NNC0165-1273 mice, suggesting the mode of action was due primarily to decreased food intake. Indeed, we saw no change to VO_2_ or VCO_2_, although there was a slight change in RER, suggesting modified energy utilization, rather than altered energy expenditure, in line with responses seen previously with PYY_3-36_ (54). Interestingly, NNC0165-1273–treated animals showed a temporary, postsurgery reduction in RER but the mechanistic explanation for this effect was beyond the scope of our studies. We also observed a near-identical reduction in lean mass (not shown) in all animals across both osmotic mini-pump studies. Because this was observed ubiquitously, we believe it be an artifact of surgical stress.

PYY_3-36_ is known to attenuate the orexigenic effects of ghrelin, which we successfully reproduced in this study. Furthermore, we showed that NNC0165-1273 also potently blocks ghrelin-induced feeding. Arcuate nucleus neurons, including NPY/AgRP neurons in particular, are known to express the Y2 receptor, so we investigated the induction of cFos in these neurones. Ghrelin potently activated arcuate NPY/AgRP neurones, as has been demonstrated before (55–58), and this effect was blocked by both PYY_3-36_ and NNC0165-1273.

Although this demonstrates the NNC0165-1273 blocks the actions of ghrelin in the same way as PYY_3-36_, it does not prove that these actions are necessarily mediated via the arcuate nucleus. To investigate this more directly, we transfected arcuate AgRP neurones with stimulatory DREADD (hM3Dq); when administered with CNO, mice ate voraciously for a number of hours, as has been seen before (35, 59). However, by preadministering NNC0165-1273, we were able to attenuate this feeding response. This fits with the knowledge that NNC0165-1273 is selective for Y2, which is known to be an autoinhibitory receptor on NPY/AgRP neurones. However, to demonstrate that NNC0165-1273 is acting on these neurones and not downstream, or even in a parallel pathway, we also used patch-clamp electrophysiology to record the electrical response of arcuate NPY neurones to NNC0165-1273 administration. We saw a potent hyperpolarization and firing inhibition in response to NNC0165-1273, indicating that the analog is able to act directly on NPY/AgRP neurons.

In summary, our data show that the more selective PYY_3-36_ analog, NN0165-1273, improved on the beneficial actions of PYY_3-36_ while avoiding adverse effects. Further, we were able to specifically block a known orexigenic pathway, and show cell autonomous effects in the arcuate nucleus. Although we have not exhausted every possibility, we have demonstrated a mechanism of action for NNC0165-1273 directly on NPY/AgRP neurones, which is important knowledge for any potential therapeutic use. Indeed, it may be important for future therapies to have limited, well-defined actions, which might be useful in combination therapies, providing potent outcomes at low doses resulting from synergistic activities. Therefore, if we can ensure a long half-life and relatively easy administration, NNC0165-1273, or a derivative thereof, could be a powerful addition to any future obesity therapy toolbox.

## Abbreviations:

aCSF: artificial cerebral spinal fluid
BSS: behavioral satiety sequence
CNO: clozapine *N*-oxide
DIO: diet-induced obese
DREADD: designer receptors exclusively activated by designer drug
GFP: green fluorescent protein
K_i_: inhibitory constant
NPY/AgRP: neuropeptide Y/agouti-related peptide
PYY: peptide YY
SC: subcutaneous
ZT: Zeitgeber time
